# Prevention of perioperative atrial fibrillation with beta blockers in elderly patient during abdominal surgery

**DOI:** 10.1186/1471-2482-13-S1-A34

**Published:** 2013-09-16

**Authors:** Gennaro Pagano, Dario Leosco, Nicola Ferrara, Nicola Rocco, Corrado Rispoli, Loredana Iannone, Serena Testa, Rita Compagna, Antonello Accurso, Bruno Amato

**Affiliations:** 1 Department of Translational Medical Sciences, Federico II University of Naples, Naples, Italy; 2 “Salvatore Maugeri” Foundation, IRCCS Scientific Institute of Telese Terme, Benevento, Italy; 3 Department of Biomedical, Surgical and Dental Sciences, University of Milan, Milan, Italy; 4 Department of General Surgery - ASL NA1, Cardinale Ascalesi Hospital, Naples, Italy; 5 Department of General Surgery, Fatebenefratelli Hospital, Benevento, Italy; 6 Department of General, Geriatric, Oncologic Surgery and Advanced Technologies, Federico II University of Naples, Naples, Italy

## Aim of the study

To investigate whether pharmacologic prophylaxis with Beta Blocker Carvedilol reduce perioperative Atrial Fibrillation (AF) in elderly patients during abdominal surgery.

## Background

Peri-operative AF is the consequence of stress activation of Sympathetic Nervous System (SNS) following surgery [1,2]. The incidence of AF increase with the aging and in presence of comorbidities [3-5]. Clinical consequences include reduced cardiac output, lengthened hospitalization, an increase in the risk of cerebral thromboembolism and in many cases the need for systemic anticoagulation [6]. SNS overactivation could be treated with Beta Blockers and may reduce the occurrence of AF [7-10].

## Methods

A prospective, randomized, single-blind, controlled pilot study in patients undergoing abdominal surgery (right emicolectomy, sigmoidectomy and anterior rectal resection) was conducted. 22 elderly patients (70 years old and over) were randomized to control (ctr) (n = 13) and Carvedilol (carv) (n = 9) groups. Treatment was received only during and 36 hours after surgery in the carv group.

## Results

The occurrence of AF were 3 in ctr group and in 0 in carv group (p < 0.0001). No statistical differences were present in demographic and clinical characteristics between two groups. No correlation was found between age and the incidence of AF..

## Conclusions

In the elderly patients underwent abdominal surgery, the reduction of SNS with Beta Blocker Carvedilol should be useful to reduce the occurrence of peri-operative AF and, consequently, to reduce the impact of AF cardiovascular complications.
